# Dentistry and Interoperability

**DOI:** 10.1177/00220345221100175

**Published:** 2022-06-10

**Authors:** N.M.R. Rajkumar, M.R. Muzoora, S. Thun

**Affiliations:** 1 Core Facility Digital Medicine and Interoperability, Berlin Institute of Health at Charité–Universitätsmedizin Berlin, Berlin, Germany

**Keywords:** artificial intelligence, big data, digital dentistry, international standards, standardization, Structured Data Capture (SDC)

## Abstract

Information has become the vital commodity of exchange in recent decades. Medicine is no exception; the importance of patient information in the digital form has been recognized by organizations and health care facilities. Almost all patient information, including medical history, radiographs, and feedback, can be digitally recorded synchronously and asynchronously. Nevertheless, patient information that could be shared and reused to enhance care delivery is not readily available in a format that could be understood by the systems in recipient health care facilities. The systems used in medical and dental clinics today lack the ability to communicate with each other. The critical information is stagnant in isolated silos, unable to be shared, analyzed, and reused. In this article, we propose enabling interoperability in health care systems that could facilitate communication across systems for the benefit of patients and caregivers. We explain in this article the importance of interoperable data, the international interoperability standards available, and the range of benefits and opportunities that interoperability can create in dentistry for providers and patients alike.

## Introduction

Data have become the vital commodity of exchange in recent decades. The volume of data collected and stored is enormous and increasing. Users and organizations are finding ways to make use of the data, learning the science of data management to accomplish their goals. There are several terms from computer scientists, such as “big data,” “machine learning,” “deep learning,” and “artificial intelligence” (AI), to define and differentiate the technologies involving data. All these newer technologies are being successfully utilized in astronomy, retail markets, automobiles, social media, web search engines, and even politics (Murdoch and Detsky 2013). The costs of using and storing data are reducing, and it is considered an inexhaustible resource (Schwendicke and Krois 2022). Estimates indicate that health care data will soon attain the levels of zettabytes and even yottabytes (Glick 2015). Almost 90% of universal data and 60% of medical data are still unstructured and text based (Malmasi et al. 2017; Adnan et al. 2020). It is fundamental to understand that data are useless when they cannot be read, retrieved, analyzed, deciphered, and reused (Obermeyer and Emanuel 2016). Furthermore, medical data can be useful only if made into meaningful information. The first step in the process of meaningfully transforming the data is to make the data structured so that they are readable by humans and computers. Another barrier is the seamless communication of these data among multiple systems and organizations, as the recorded data are often hidden in isolated silos and incompatible systems, consequently making the data less useful (Lehne et al. 2019).

### Electronic Health Records

Even though technological barriers, perceived lack of relevance, and high costs have limited the adoption of electronic health records (EHRs) in medicine and dental practices, there has been a gradual increase in the usage of health information technologies with expectations to improve the quality, accessibility, affordability, safety, and equity of health care (Schwendicke and Krois 2022). Procurement of EHR systems needs crucial analysis according to the clinicians’ workflow in the health care facility and involves several thousands and at times millions of dollars (Lancet Editorial 2018). Yet, many such EHR systems promote free-text data entry, rather than a structured form facilitating stakeholders to store similar data in numerous locations, thereby making the data inconsistent and useless (Song et al. 2013). These data in these isolated information systems need to be cracked and made available through secure access for reuse (Schwendicke and Krois 2022). Dentistry lags in the adoption of health information technology (IT) systems, but the initial move toward the structured and secure digitization is to procure an electronic dental record system. The foundation of health data management composed of recording, storage, and exchange is the implementation of an international health care data standard in electronic dental records (Joda et al. 2019). During the procurement, the primary checkpoint to consider is to analyze whether the systems are interoperable and compatible with current international health care data standards. The Meaningful Use initiative came into practice in the United States, which encouraged and provided incentives to promote the use of interoperability-enabled EHRs. This drove the electronic medical record manufacturers to adopt international health care interoperability standards into their EHRs, while still some stood behind (Kalenderian et al. 2013). In Europe, an approach was taken without incentives but as a community to follow the FAIR principles (findable, accessible, interoperable, and reusable). The European Commission (2017) led the course by providing an implementation roadmap for the European Open Science Cloud, setting out the actions needed to develop shared resources to define the operational guidance and methodologies for applying the FAIR principles.

### Practice-Based Research

As the “evidence-based medicine” movement came into existence, it demonstrated that scientific analysis is above expert opinions and testimonials. As compared with all other domains (e.g., automobile and aviation industry), medicine and dentistry have been ahead for decades in evidence recognition and analytic decision making (Murdoch and Detsky 2013). Using EHR data for research brings forth increased efficiency, lowered costs, potential for providing critical information for clinicians, comparative effectiveness, and progress in epidemiologic and further research fields. There has been significant approval and usage of electronic dental records in dental practices due to the adoption of computers in the digital age. In recent times, the use of electronic dental record data has become progressively more interesting among the dental research community, but there have also been limitations in the current electronic dental records. The first limitation is the inability to communicate the information to other systems. Second, there is a limitation through the inconsistency of the communicated information, being incomplete, inaccurate, and missing. The DMF index (decayed, missing, and filled) recorded by dentists in Finland varied greatly, resulting in errors and inconsistencies in the collected data. In a comparative study on the relationship between dental caries in children and the Apgar score, the primary Apgar score variables were missing for a few participants, posing a hurdle for statistical analysis (Song et al. 2013). Usage of international health care data standards will provide greater uniformity in the collected data and better aggregation for further analysis and learning. Creating possibilities for health care workers to collect standardized health care data is a necessity to finely understand the trend patterns of diseases and the treatment outcomes (Joda et al. 2019).

## Interoperability

Interoperability can be broadly defined as “the ability of two or more systems or components to exchange information and to use the information that has been exchanged” (Geraci 1991). Interoperability can be further differentiated by components, layers, or levels. The differentiation ranges from lower-level technical components to higher-level organizational components. A brief description of the technical, syntactic, semantic, and organizational aspects of interoperability follows (Lehne et al. 2019).

### Technical Interoperability

Technical interoperability is the foundation for any information exchange. The moving of data from system A to system B is technical interoperability regardless of the distance and domain. It moves the data without knowing the meaning or format of the data (Benson and Grieve 2021). For a meaningful health data exchange, semantic and syntactic interoperability is essential with technical interoperability (Lehne et al. 2019).

### Syntactic Interoperability

**Figure 1.**
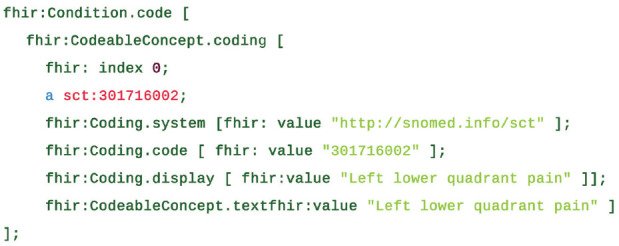
An example of a patient complaint in the Fast Health Interoperability Resource, “condition,” with coded concept in SNOMED CT, “left lower quadrant pain,” enabling machine and human readability (Health Level Seven International 2019).

Syntactic interoperability is defining the format and structure of the data. The idea of the exchange of structured health data is backed by international standards development organizations such as Health Level Seven International and Integrating the Healthcare Enterprise (IHE), which define health IT standards and their use across health care systems. Health Level Seven International’s (2019) Fast Health Interoperability Resource (FHIR) is an emerging communication standard for health data being widely adopted by the health care industry (Fig. 1). FHIR has >140 resources that are common health care concepts used for accessing and exchanging data through modern web solutions. A similar drive to initiate the structured exchange of health data is openEHR. Health care professionals themselves can define the clinical content using archetypes (Lehne et al. 2019).

### Semantic Interoperability

Semantic interoperability is the core of the health care domain, dealing with medical terminologies, nomenclatures, and ontologies. They finely define the semantics of health data after the commonly used standards, such as FHIR and openEHR. Semantic interoperability protects the meaning of medical concepts in the shared health data environment, enabling understanding in humans and computers. There has been a steady development in the area. The SNOMED CT terminology (Systematized Nomenclature of Medicine-Clinical Terms) has almost 340,000 medical concepts, which include clinical findings, procedures, substances, organisms, and body structures (Fig. 1). It is considered an all-purpose medical and health care language for advancing semantic interoperability. Logical Observation Identifiers Names and Codes is another domain-specific terminology for laboratory observations. Additionally, there are the Identification of Medicinal Products for medicine, the Hugo Gene Nomenclature Committee for genes, and the Human Phenotype Ontology for phenotypic ontologies. Use of these semantic standards in combination will ensure that health data are clearly structured and unambiguous (Lehne et al. 2019).

### Organizational Interoperability

The highest level of interoperability is organizational interoperability, which deals with organizations, legislations, and policies. For a seamless exchange of health data and implementation of the aforementioned standards, it requires the organizations and regulatory bodies to understand the importance of interoperability. The aim is to motivate health care professionals to utilize the available technologies to further improve patients’ health, although this demands common processes and workflows that equip health care across institutions. It can be achieved through incentives, funding for researchers, and, if needed, rigorous legal regulations (Lehne et al. 2019).

## Opportunities and Benefits

The benefits of enabling interoperability are endless; it can be implemented and be beneficial in almost all its applications. There are some barriers to these opportunities and benefits. First, there have been several other locally developed and implemented interoperability standards that only partially fulfill the goals of interoperability: EZCodes dental diagnostic terminologies (later changed to Dental Diagnostic System; Obadan-Udoh et al. 2017), diagnostic terminologies in dentistry (Obadan-Udoh et al. 2017), and SNODENT (Systematized Nomenclature of Dentistry), a subset of SNOMED CT (Tokede et al. 2013). These terminology systems are not recognized internationally and do not cover the whole scope of medical terminologies, just the dental science. Second, because of the bespoke interfaces of the EHR systems, some institutions get their interfaces customized where there arises a need for a proprietary standard (Benson and Grieve 2021). Even though the codes can be mapped to international standards, a barrier prevails, stopping users from embracing the complete freedom, reliability, and benefit of the available international standards. This section briefly elaborates some of the benefits from the implementation of international interoperability standards.

### Cost-Benefit

Current EHR system manufacturers create their own proprietary and nonstandardized protocols, allowing the systems to exchange data only among themselves, locking in the health care provider and making interoperability with other systems difficult (Lancet Editorial 2018). They are fragmented and have limited interoperability. This has given rise to third-party markets where necessary bridging applications are designed to fill the gap among individual systems but at an additional cost. EHR manufacturers tend to lock health care providers into their proprietary systems for financial benefits (Lancet Editorial 2018). By the use of interoperable systems, the cost and benefits received from data reusability are enormous (Joda et al. 2021). A drag in the development of the digital health care industry is evident due to the lack of interoperability: an estimated $36 billion in time wasted with manual reentering of data, conventional transmission of data, and repetitive research due to missing or unavailable data (Lancet Editorial 2018).

### Data Exchange

**Figure 2.**
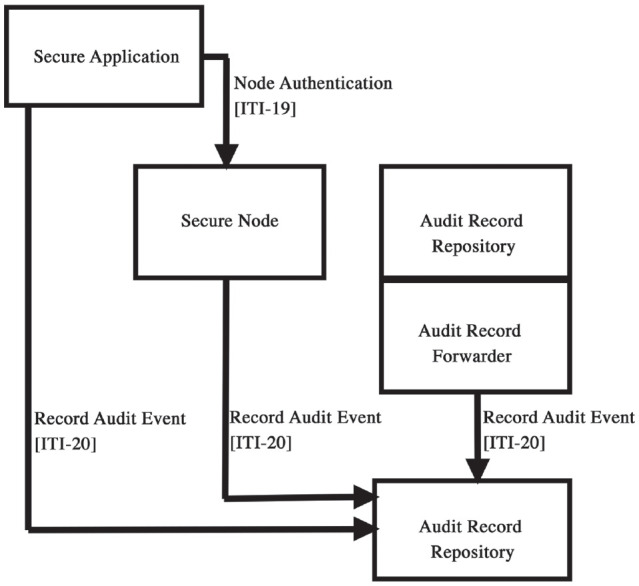
The diagram for Integrating the Healthcare Enterprise’s Audit Trial and Node Authentication profile shows each actor and transaction (IHE International 2022).

The rise in digital health technologies brings concerns that health care workers must spend more time with data entry and documentation and not with their patients. In reality, interoperable EHRs can relieve the burden of data entry and cumbersome documentation processes, facilitating health care workers to concentrate on their patient care (Perlin 2016). Interoperable data are scarce to find, and when large data sets are needed for extensive research on rare diseases, precision medicine, or drug development, exchanging health data among different health IT systems is of absolute importance. With rare diseases, a health care institution handles a handful of cases and needs a better understanding to improve diagnosis and management, for which seamless data exchange is a prerequisite. The first exchange of common data models within European countries was in 2019. This shows the possibility of interoperable data exchange among a wider community (Lehne et al. 2019). The capability of data can be realized only when they are made available across clinical, scientific, national, and international borders (Jones et al. 2017). As an example in relevance, a boy died in 2011 after systematic abuse. There were several visits to the physician’s practice, primary health care center, and emergency department and a visit by the health care professional. The data from each instance were locked in isolated silos and not presentable at the time of need (Jones et al. 2017). Data mining from multiple EHRs is a possible solution for lifesaving patient care management (Glick 2015). The security concerns in the health care area have been on the rise in the last few years, and these concerns can be solved with international initiatives such as the IHE (Office for Civil Rights 2022). The IHE has several profiles to standardize and regulate communications among computer systems. Namely, the International User Authorization profile and the Audit Trail and Node Authentication profile can provide secure transaction of information between systems and organizations (Fig. 2). Use of such international standards and frameworks can simplify the requirements for convenient and secure data transfers (IHE International 2022).

### Data Analytics

More than sophisticated analytics or complex AI algorithms, making the right information available at the right time is a lifesaver (Lehne et al. 2019). Unstructured data can be made interoperable through complex algorithms, which map the attributes to a common fixed format (FHIR). This needs efficient machine learning capabilities, and it is known as natural language processing (Liang et al. 2019). It could include an AI algorithm programmed to find patients with diabetes from an unstructured text, but it could also include patients with family histories of diabetes into the list. These errors are then difficult to find, and in the large volume of data, it is complex to anticipate, detect, and correct all the errors. Such problems are even more prone in artificial neural networks and deep learning algorithms. It is always better to use data with clear structure and unambiguous semantics; otherwise, modern AI algorithms can do more harm than good (Lehne et al. 2019).

AI and large-scale data analytics are often combined with the term *big data*. Recently this has been increasingly transforming medicine and health care. They are dependent on an expanding amount of health data, and these technologies need maximum input from various sources for a comprehensive analysis (Lehne et al. 2019). Big data have the capability to establish an observational evidence base for clinical questions in other ways that would not be possible.^1^ Predictive analytics can be envisioned with all the available metrics for an unexpected event; it can warn the patients and the physicians in time (Topol et al. 2015). Prognostic models such as the APACHE score (Acute Physiology and Chronic Health Evaluation) and SOFA score (Sequential Organ Failure Assessment) could be easily drawn from EHRs to make exceptional predictions (Obermeyer and Emanuel 2016).

Health care professionals can soon decode and interpret patient data synchronously, which could include an oral microbiome that can express the state of health or the disease of the patient. This will be possible through the collected genomic, proteomic, transcriptomic, and metabolomics data, to be also used in pharmacogenomics, precision medicine, and personalized oral care. These advanced technologies have the ability to detect minute processes and can generate data that can give rise to new therapeutic agents. Translational genomics has already helped in identifying the subtypes of cancers, eventually providing for the improved treatment (Glick 2015).

Six *V*’s are commonly used to explain the concept of big data: value (relevance of the data), variability (evolution and seasonality of the diseases), variety (data from various sources), volume (quantity of data and high-throughput technologies), velocity (speed of processing and generation of new data), and veracity (quality of data). Out of the 6 variables, 4 involve data, and when they are structured, big data can transform health care (Glick 2015).

### Dental Research

There has been a steady growth in dental research, from innovations to transformations in the workflow of dentistry. The digital workflow in dentistry has paved a way for an almost complete revamp of conventional workflow to a complete digital dentistry. Digital radiographs, oral scans, CAD/CAM designing, milling, and 3-dimensional printing have been the innovations, but they are all proprietary and are completely independent within themselves. These innovations produce enormous data that could enhance dentistry overall. With the existing data (if not in silos), the design of a prosthodontic oral cavity could be entirely automated using AI algorithms and machine learning.

Devices known as the *internet of things* have come up in the innovation market of dentistry. Removable mouth guards that measure glucose and uric acid concentrations in saliva could produce groundbreaking results with the data (Schwendicke and Krois 2022). Nowadays, toothbrushes record enormous information, but most consumer brands do not have interoperable data and the data are not open, restricting for reuse and benchmarking (Dwivedi et al. 2021). A geographic information system in the field of dentistry to measure water fluoride coverage could further help dentists in their decision making and extend more accessible dental care services (Schwendicke and Krois 2022). If all these real-world data are available and interoperable, they could be used for large-scale observational studies at all levels to address epidemiologic and public health concerns (Lehne et al. 2019).

## Conclusion

There is a definite need in dentistry for the implementation of international interoperable standards as well as the contribution to the standards. The future depends on interoperable data: to utilize the ultimate potential of AI and big data, to improve communication between dentists and hospitals, and for an affordable research environment. Efforts from individual to governing authorities are crucial for this transformation. Making data meaningful and overcoming barriers between individuals and organizations through interoperability will raise the knowledge and care delivery among health care workers (Lehne et al. 2019).

## Author Contributions

N.M.R. Rajkumar, contributed to conception and design, drafted the manuscript; M.R. Muzoora, contributed to data acquisition, critically revised the manuscript; S. Thun, contributed to data acquisition, analysis, and interpretation, critically revised the manuscript. All authors gave final approval and agree to be accountable for all aspects of the work.
